# NMR studies of polymeric sodium ion conductors—a brief review

**DOI:** 10.3389/fchem.2023.1296587

**Published:** 2023-11-09

**Authors:** Allen Zheng, Steven G. Greenbaum

**Affiliations:** Hunter College of CUNY, New York, NY, United States

**Keywords:** sodium batteries, polymer electrolytes, NMR, structure, transport

## Abstract

Sodium has long been considered an alternative active battery cation to lithium because of the chemical similarity and the overwhelming natural abundance of Na compared to Li. In the “early days” of poly (ethylene oxide) (PEO) and alkali metal salt complexes proposed as polymer electrolytes, studies of Na-salt/PEO materials were nearly as prevalent as those of lithium analogues. Fast forwarding to the present day, there is growing interest in sodium battery chemistry spurred by the challenges of continued advancement in lithium-based batteries. This article reviews the progress made in sodium-based polymer electrolytes from the early days of PEO to the present time. Other polymeric electrolytes such as gel polymer electrolytes (GPE), including formulations based on ionic liquids (ILs), are also discussed.

## Introduction

There is an urgent need to progress society beyond a reliance on fossil fuels and into a sustainable future built on renewable energy. As renewable energy storage shortages become apparent across a multitude of industries, the electrolyte battery will play a key role in the development of energy storage systems to maintain steady energy flow to consumers. In comparison to fossil fuels, which store energy within their chemical bonds, renewable energy sources require energy storage systems to maintain a capacity large enough to meet consumer demands since the energy sources—namely, wind, solar, and hydroelectric—rely on inconsistent natural phenomenon and provide intermittent power. Common energy storage systems include pumped hydroelectric systems, hydrogen fuel cells, and chemical batteries. As the demand for storage systems increases, research on battery systems is required to scale increased capacity needs (Leonard and Michaelides, 1970). Common lithium-ion battery (LIB) packs cost around $1,100/kWh in 2010, proving too expensive to scale to required demands. In 2020, this cost has dropped to $156/kWh, opening the market for large scale grid storage and electric automotive industry (Trahey et al., 2020). These systems must still be improved to allow the full transition away from fossil fuels systems.

Currently, LIBs are one of the most popular and well-studied electrolyte battery types due to a plethora of advantageous properties of lithium and associated systems. Lithium is the lightest (0.534 g cm^−3^) and most electronegative (∼−3.0 V vs. SHE) metal, allowing LIBs to attain high cell voltages and energy density. (Xu, 2004) Many rechargeable LIBs provide long cycle life, quick charge and discharge capabilities, and stable long-term voltages (Xu, 2004; Goonan, 2012). Due to these key features, LIBs play a major role in the battery market, powering many hand-held appliances, hybrid and fully electric automotives, and grid-storage systems (Goonan, 2012).

Despite these advantages, several critical disadvantages plague a future reliance on Li-ion batteries. Growing demand for LIBs has greatly increased mining operations in lithium-rich areas of Bolivia, Chile, Argentina, and Australia. These mining operations are increasingly fraught with environmental issues. Some forms of lithium mining require large amounts of water, resulting in the extraction of groundwater in already arid areas, which leads to alteration of the local geological and biological landscape. Due to the high mobility of lithium and the large number of chemical substances used in the mining process, areas surrounding lithium mines are at risk of chemical pollution. These effects of water scarcity, habitat destruction, and pollution in addition to the expansion of mining sites lead to forced migration of local populations. Local labor practices favor the State and national and foreign companies, while sacrificing the rights and health of local laborers (Agusdinata et al., 2018). Once on the market, LIBs prove volatile and dangerous during operation and storage. LIBs failures have been the culprit of fires and explosions in many appliance, automotive, and grid-storage systems (Chen et al., 2021). Safety concerns for LIBs arise from easy growth of dendrites and other defects on electrode interfaces which increases the likelihood of electric shortages and high flammability of liquid electrolytes (Guo et al., 2022). Solid electrolytes and polymer electrolytes improve on these drawbacks. For these reasons, significant research is dedicated to lithium-alternative polymer electrolyte battery types which utilize components with reduced environmental and socio-economic burdens while improving safety requirements.

The sodium-ion battery (SIB) is one candidate to replace LIBs. SIBs—designed on the same working principles as those for LIBs—are attractive due to the potential lower costs, and more stable chemistries associated with sodium. Sodium is a highly abundant element worldwide with well-established global production industries. These factors could greatly reduce the monetary, environmental, and socio-economic costs of battery technologies. SIBs could offer several safety advantages, including zero voltage storage, high thermal stability at high temperatures, and lower thermal runaway rates. However, several hurdles are present, including poor dynamic properties of larger and heavier sodium ions, high solubility of solid electrolyte interphase (SEI), and poor electronic/ionic conductivity of cathode materials (Yang et al., 2020a).

Interfacial behaviors between polymer and other electrolytes and electrodes are of particular interest in the development of novel SIB systems. Dendritic growth of the SEI due to nucleation of electrode materials in contact with electrolytes during repeated charge and discharge cycles can lead to decreased cell potential and battery performance, and can even lead to electrical shortage, often to disastrous effect in high load systems. Finding chemistries to mitigate nucleation of dendrite sites and improve electrolyte/cathode contact to provide stable interfaces and improve electronic performance during cycle life is necessary.

Research on non-aqueous polymeric sodium electrolytes is underway to address these issues. The similarities between lithium and sodium as the first two members of the alkali series allow researchers to directly compare properties and performance of sodium polymer electrolyte chemistries derived from existing lithium polymer electrolyte chemistries, though several novel chemistries are studied using sodium. The most common polymer matrixes include poly (ethylene oxide) (PEO), poly (methyl methacrylate) (PMMA), polypropylene carbonate (PPC), polyacrylonitrile (PAN), among many others (Zhang et al., 2020).

Poly (ethylene oxide) (PEO) is an amphiphilic copolymer and is the most commonly studied polymer host in rechargeable battery electrolyte chemistries. The amphiphilic structure of PEO creates differences in solubility and expresses various phase behaviors (Li et al., 2014). Pure PEO is partly crystalline with a melting temperature of 333 K and a glass transition temperature of 213 K. Additions of various salts can significantly increase ionic conductivity at temperatures above the melting point of PEO (Munshi and Owens, 1988).

Nuclear magnetic resonance (NMR) spectroscopy is a high-resolution technique used to study the responses of nuclear spins in a liquid or solid bulk sample within a strong static magnetic field to radio frequency pulses. NMR allows detailed analysis of lattice structures and molecules, intermolecular interactions, and important dynamic properties of target nuclei. Common nuclei studied in battery research include ^1,2^H, ^6,7^Li, ^13^C, ^19^F, ^23^Na, and ^27^Al. NMR involving ^23^Na can require special consideration due to the relatively strong quadrupolar interactions arising from this spin 3/2 nucleus. NMR spectra targeting nuclei with these properties result in broadened lines due to short T_2_’s. These T_2_’s can be on the shorter end of the NMR timescale, making diffusion difficult or impossible. In some cases discussed later, NMR experiments targeting the anion – typically by ^19^F NMR – are used in such cases where ^23^Na diffusion NMR is not possible.

Common techniques that will be discussed include magic angle spinning (MAS) experiments for high-resolution study of local structure of solids to narrow broad NMR lines caused by dipolar, chemical shift anisotropy, and quadrupolar interactions found in solid samples, and pulsed field gradient (PFG) experiments used to study dynamic properties of electrolytes by targeting nuclei found in cations and anions to separately analyze ion mobility and estimate transference numbers (Abragam, 1994). Information gathered from 1D NMR experiments can provide insight on bonding structure in electrolytes and SEI materials via chemical shift. These techniques can provide insight on coordination structure and ion pairing in electrolytes. Additional techniques can involve analysis of variable temperature, linewidth, transference numbers, and more. In particular, these techniques in combination with other types of measurements are useful tools to determine ion transport properties of battery electrolytes that are favorable for sodium polymer electrolytes which can compete with current lithium polymer electrolyte standards. This paper will review some of the history and current state of research on sodium-based PEO and other polymer electrolyte batteries through the analysis of nuclear magnetic resonance techniques used to investigate structures and transport dynamics.

## Results

### PEO

The earliest work demonstrating fast ion transport on PEO-based electrolytes by Fenton et al., achieved high conductivities on the order of 10^−4^–10^–5^ S cm^−1^ at room temperature with various compositions of PEO and a 50% epoxidized natural rubber (ENR50) and LiCF_3_O_3_ salts (Fenton et al., 1973). Conductivity results showed the transition of PEO from semi-crystalline state to amorphous state at increased LiCF_3_SO_3_ concentration. The following is a brief review of some of the recent studies on PEO-Na salt systems in the 50 years since the work of Fenton et al., in 1973.

**FIGURE 1:**
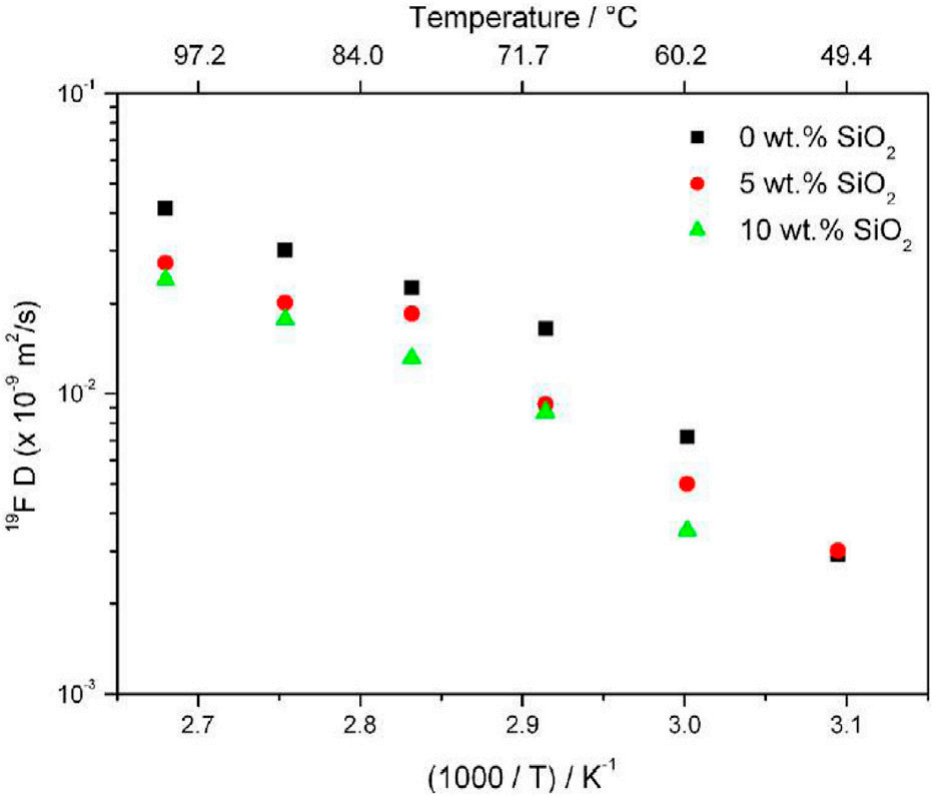
Anion self-diffusion plot in PEO_20_:NaTFSI with 0, 5, and 10 wt% of SiO_2_ from ^19^F PFG NMR results. Reproduced with permission from Ref. (Serra Moreno et al., 2014), Journal of Power Sources, vol. 248, 2014.

Moreno et al., of the Sapienza University of Rome investigated sodium bis (trifluoromethanesulfonate) imide (NaTFSI) and PEO complex with various PEO:NaTFSI ratios with the addition of ceramic fillers SiO_2_ with 0, 5, and 10 wt% of silica filler (Serra Moreno et al., 2014). Due to strong quadrupole coupling of ^23^Na, spin-lattice relaxation times (<1 m) are often too short for the standard pulsed field gradient (PFG) NMR. Therefore, cation diffusion and dynamics were studied based on anion dynamics targeting ^19^F self-diffusion results. For PEO_20_: NaTFSI, ^19^F PFG NMR experiments revealed lower self-diffusion rates of anions with increasing SiO_2_ concentration, with a more significant decrease between 0 and 5 wt% than 5 and 10 wt% (Figure 1). The conductivity of the 5 wt% filler sample is similar to that of the 0 wt% filler sample, indicating a higher cation transference number. This is confirmed from transference numbers estimated from chronoamperometric curves (Table 1).

**TABLE 1:** Transference numbers for Na^+^ cation in PEO_20_:NaTFSI with 0, 5, and 10 wt% of SiO_2_ from chronoamperometric curves. Reproduced with permission from Ref. (Serra Moreno et al., 2014), Journal of Power Sources, vol. 248, 2014.

EO:NaTFSI	$t_{Na}^{+}$
20:1	0.39
20:1, 5 wt% SiO_2_	0.51
20:1, 10 wt% SiO_2_	0.48

**FIGURE 2:**
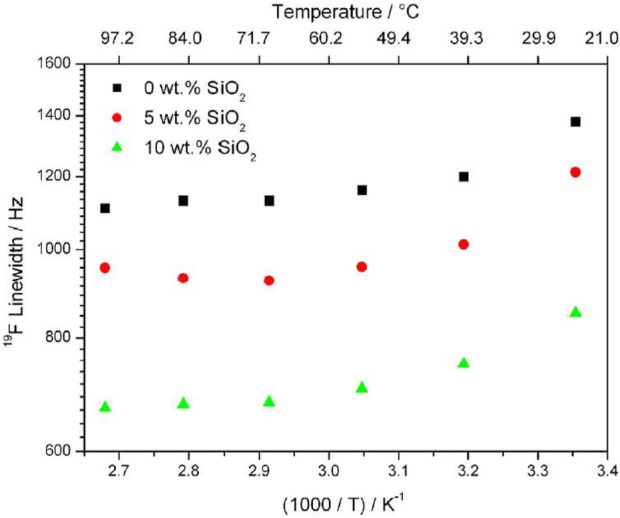
Linewidths of ^19^F in PEO_20_: NaTFSI with 0, 5, and 10 wt% of SiO_2_ from ^19^F 1D NMR results. Reproduced with permission from Ref. (Serra Moreno et al., 2014), Journal of Power Sources, vol. 248, 2014.

Variable temperature 1D NMR results show decreasing linewidth of ^19^F with increasing temperature and this effect is increased with increasing filler concentration (Figure 2). Samples with higher filler concentration also exhibit narrower NMR linewidths, though this is attributed to rapid rotation of the CF_3_ group about its symmetry axis, and not increased cation transport.

**FIGURE 3:**
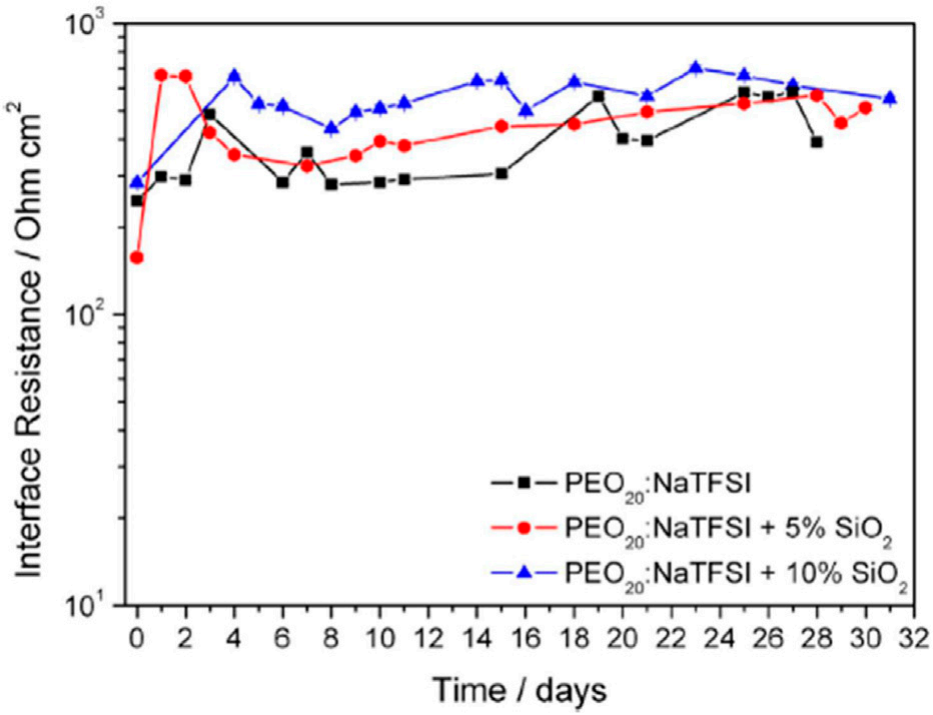
Interfacial resistance time evolution of PEO_20_:NaTFSI added by n wt% SiO2 (*n* = 0, 5, 10). Reproduced with permission from Ref. (Serra Moreno et al., 2014), Journal of Power Sources, vol. 248, 2014.

The polymer matrix at higher Na salt concentrations was sticky and rubber-like, signaling improved electrode interfacial contact, while sacrificing mechanical efficiency. Electrode interfacial performance was determined through impedance analysis. Symmetrical Na (s)/polymer/Na (s) cells at 75°C ± 1°C were evaluated by applying ±5 mV amplitude signal between 100 Hz–75 kHz frequency range over the course of a month of the PEO_20_:NaTFSI added by *n* wt% SiO_2_ (*n* = 0, 5, 10) (Figure 3). No physical deterioration of the membranes was observed, as confirmed by Electrochemical Impedance Spectroscopy (EIS) analyses, however unstable impedance response was determined to be due to a continuous a formation-fracture process of the passivation layer of the metallic sodium electrode. Increased filler enhanced system resistance compared to the pristine PEO_20_: NaTFSI membrane. All membranes exhibited increased impedance over time, due to the increasing thickness of the passivation surface film on electrodes.

**FIGURE 4:**
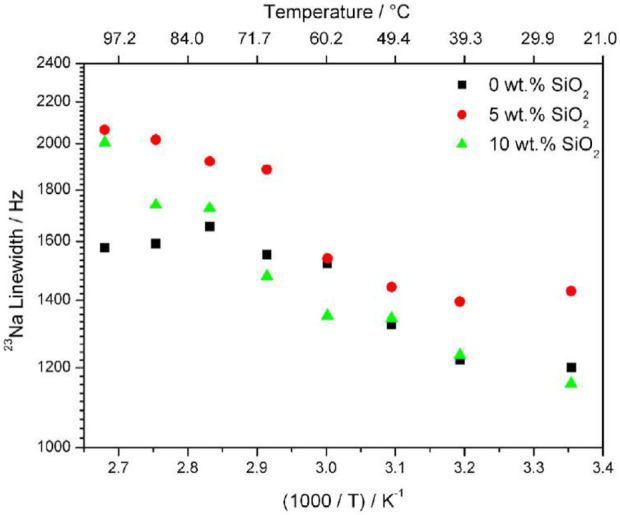
Linewidths of ^23^Na in PEO_20_: NaTFSI with 0, 5, and 10 wt% of SiO_2_ from ^23^Na 1D NMR results. Reproduced with permission from Ref. (Serra Moreno et al., 2014), Journal of Power Sources, vol. 248, 2014.

Although direct diffusion NMR measurements for ^23^Na were not possible, variable temperature 1D NMR linewidth measurements revealed a surprising increase in linewidth with increasing temperature (Figure 4). This is contrary to what is usually observed when increasing temperatures cause faster translational motion of ions and faster T_2_ relaxation, leading to narrower lines. This effect could be due to very short spin-lattice relaxation times at higher temperature caused by increased translational motion of ^23^Na ions. The samples with higher concentrations of filler exhibit a larger change in motional broadening. Conductivity measurements show a non-linear increase of conductivity with increasing NaTFSI concentration, with conductivities at 20:1 EO: Na concentrations. Through these results, the addition of filler has significant impact on the mechanical and ionic environments of the anion, though the effect of the filler on the long-range transport properties of the cation cannot be stated with the same level of certainty and must be further investigated.

**FIGURE 5:**
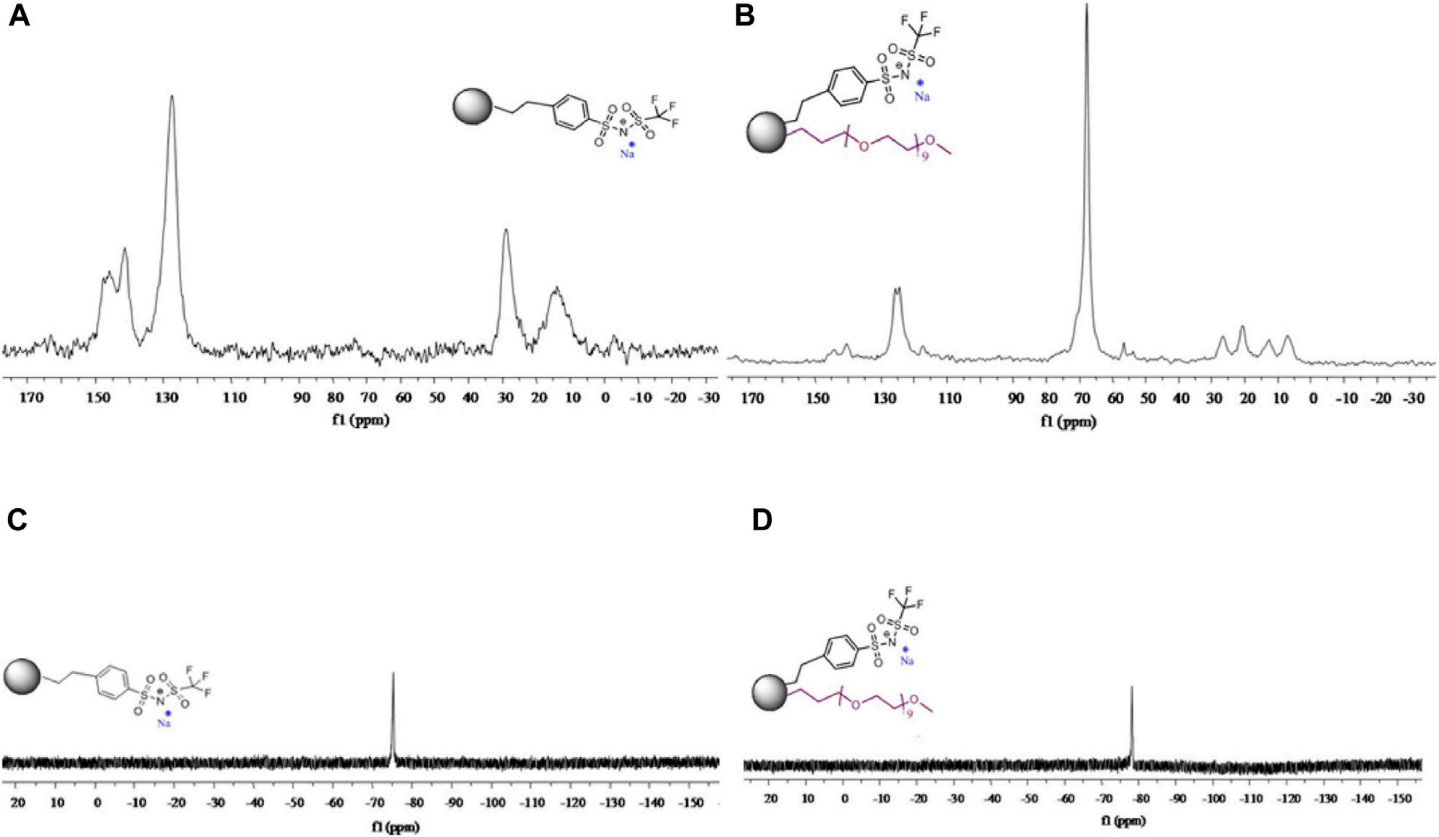
**(A)** ^13^C NMR spectra for SiO_2_-anion nanoparticles. **(B)** ^19^F NMR spectra for SiO_2_-anion nanoparticles. **(C)** ^13^C NMR spectra for SiO_2_-PEG-anion nanoparticles. **(D)** ^19^F NMR spectra for SiO_2_-PEG-anion nanoparticles. Reproduced with permission from Ref. (Villaluenga et al., 2013), Journal of Materials Chemistry A, vol. 1, no. 29, 2013.

Villaluenga et al., also studied the effects of SiO_2_ nanohybrid polymers on PEO complexes (Villaluenga et al., 2013). SiO_2_ nanoparticles were grafted with sodium 2-[(trifluoromethane-sulfonylimide)-N-4-sulfonylphenyl]ethyl and/or polyethylene glycol and PEO and polyethylene glycol dimethyl ether (PEGDME) were used as the polymer matrix with a weight ratio of 1:1 to create SiO_2_-anion and SiO_2_-PEG-Anion samples. The organic functionalization of the hybrid nanoparticles was confirmed using ^13^C and ^19^F NMR (Figure 5). Ionic conductivity as a function of sodium content was measured using EIS.

**FIGURE 6:**
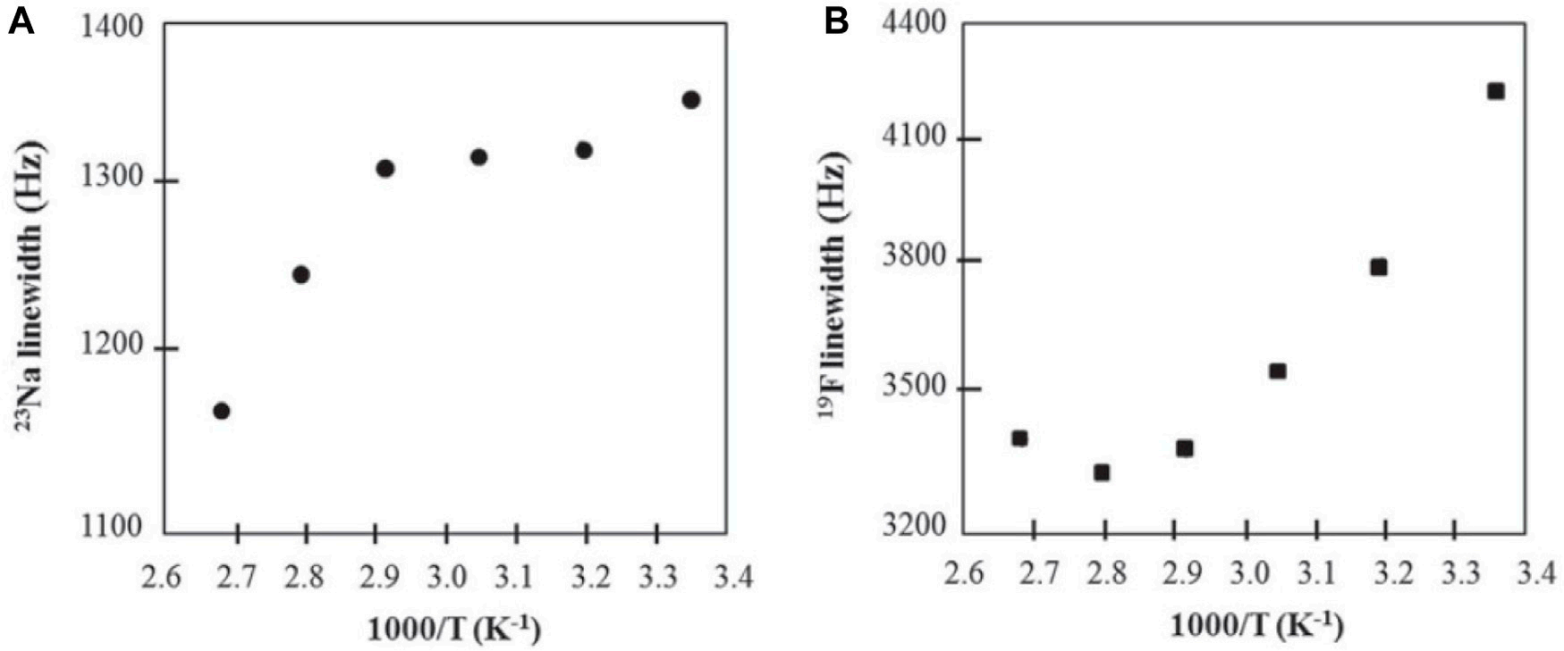
Arrhenius plots of **(A)** ^23^Na and **(B)** ^19^F NMR linewidths of SiO_2_-anion (EO/Na ∼ 10) electrolyte. Reproduced with permission from Ref. (Villaluenga et al., 2013), Journal of Materials Chemistry A, vol. 1, no. 29, 2013.

As with Moreno et al., short spin-lattice relaxation times of ^23^Na eliminated the possibility of self-diffusion measurements (Serra Moreno et al., 2014). Additionally, ^19^F PFG NMR experiments revealed no diffusive decay, implying an anion diffusion constant no higher than ∼10^−9^ cm^2^ s^−1^. SiO_2_-PEG-anion samples showed conductivities increasing with temperature and sodium concentration. For SiO_2_-anion samples, conductivity increased with temperature and a maximum conductivity was reached when the ratio of EO/Na ∼10 before conductivity decreased at higher sodium concentrations since at higher sodium concentrations, Na^+^ ions form ionic crosslinks within the polymer matrix, decreasing segmental mobility (Kumar and Hashmi, 2010). SiO_2_-anion samples were studied using ^19^F and ^23^Na variable temperature linewidth measurements (Figure 6). Linewidths for both nuclei decrease with increasing temperature and indicate a motional range similar to polyether-Na-salt polymers and the mobility range of sodium salts. These results show promise in hybrid nanoparticles for polymer electrolytes.

A study by Youcef et al., compares the transport properties of Li (TFSI-ethycellulose) and Na (FSI-ethycellulose) and PEO complexes, though lacking in ^23^Na NMR results (PAK et al., 1991). It was found that Li^+^ transference number is 0.90 while the Na^+^ transference number is 0.60, with the discrepancy possibly due to the presence of trace Na^+^ ions left after the preparation process. An electrochemical stability window of ∼4 V for Li (FSI-ethycellulose) and >4.3 V for Na(FSI-ethycellulose) was shown using AC impedance spectroscopy. Half cells of PEO/Na (FSI-ethycellulose) electrolyte with Na metal anodes and HC cathodes showed a discharge/charge capacity of 220/178 mAh/g, an initial coulombic efficiency of 80.5%, and decent cycling capability with discharge capacity approaching 100 mAh/g after 17 cycles. This preliminary Na-salt battery test shows promising battery performance considering possible optimization of electrode materials and improvement of transference number of Na^+^ ions through optimization of the preparation process.

### Other related Na electrolytes

**FIGURE 7:**
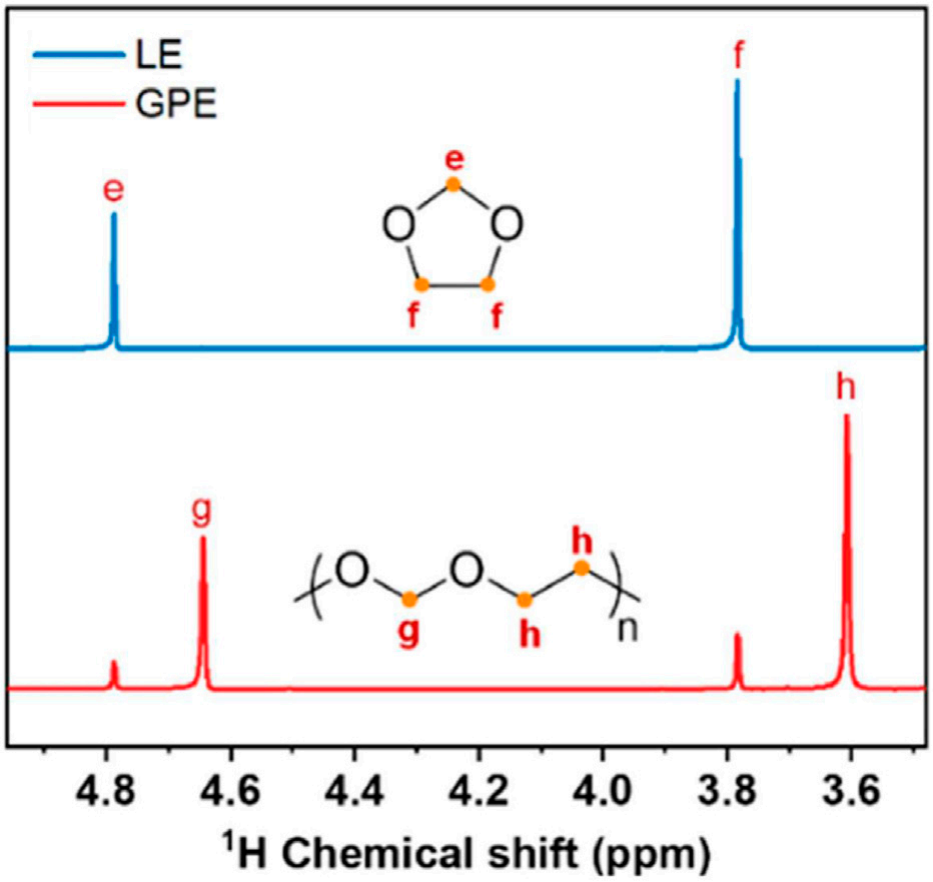
^1^H NMR comparing LE (liquid electrolyte, pristine DOL) and GPE. Reproduced with permission from Ref. (Zhang et al., 2021), Journal of Power Sources, vol. 516, 2021.

An in-situ mixed polymer electrolyte developed by Zhang et al., of Zhengzhou University involves a 4 mM concentration aluminum triflate [Al (OTf)_3_] polymerization initiator with 0.3 M NaPF_6_ salt in 2 M Na (OTF)_3_/dimethyl ether (DME), spontaneously developing into a polymerized gel when combined with dioxolane (DOL) with a 1:1 volume ratio (Zhang et al., 2021). Polymerization was confirmed through ^1^H NMR spectra showing polymerized and pristine DOL peaks (Figure 7). Peaks e and f correspond to protons in pristine DOL, and peaks g and h correspond to polymerized DOL, showing up to 86% DOL participation in polymerization. EIS measurements throughout the polymerization reaction showed a decreasing ionic conductivity over 5 h until stabilization at 3.66 × 10^−4^ S cm^−1^, showing ionic conductivity one to two orders higher than that of common polymer electrolytes such as PEO. This is in part attributed to free DME facilitating fast transport of solvated Na^+^.

**FIGURE 8:**
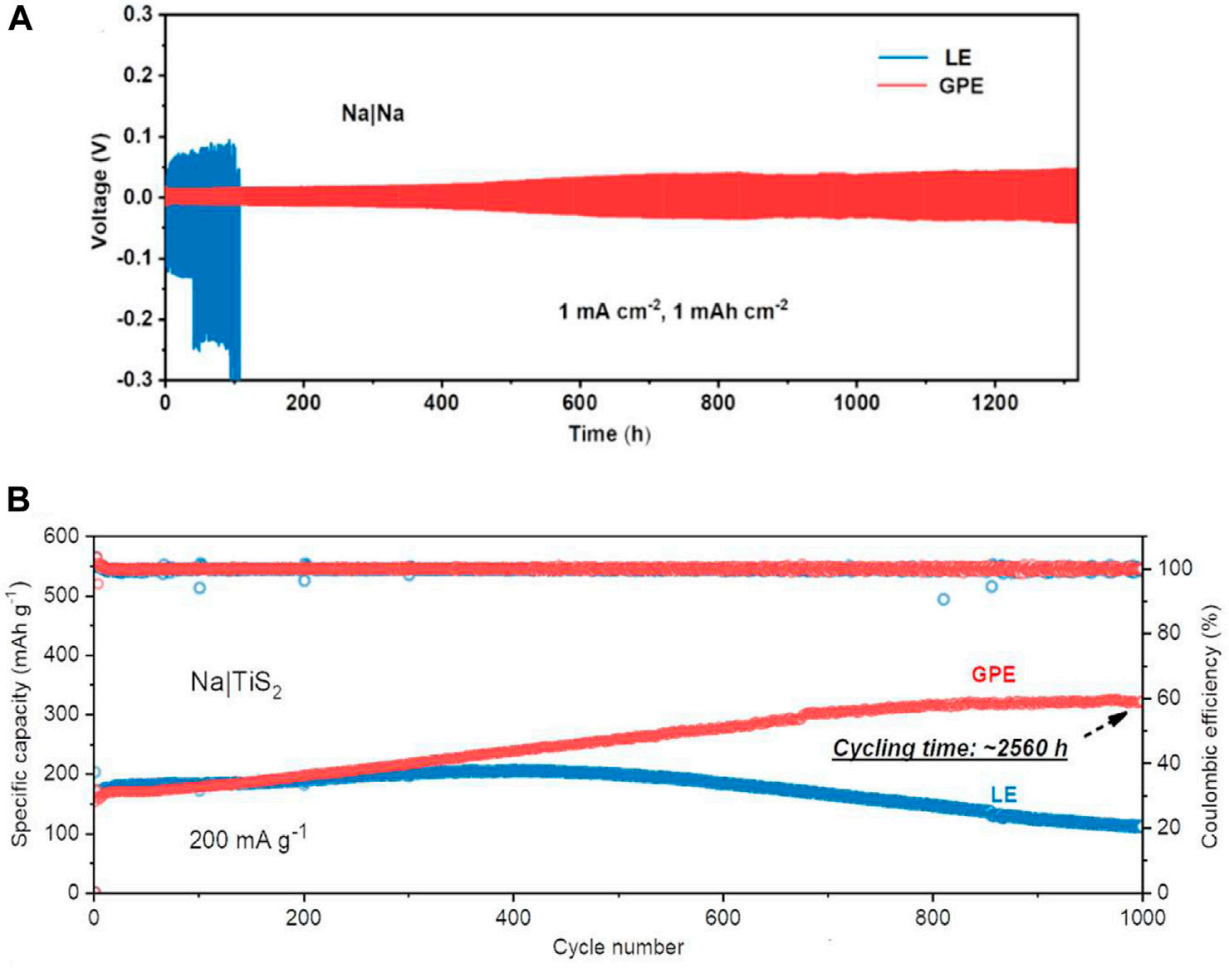
**(A)** Overpotential comparison of Na|Na cells with LE and GPE. **(B)** Capacity comparison of Na|TiS _2_ cells with LE and GPE. Reproduced with permission from Ref. (Zhang et al., 2021), Journal of Power Sources, vol. 516, 2021.

Na|Na symmetric cells constructed with LE and GPE showed greater than 0.1 V overpotentials after 45 h for LE and stable cycles for 1,300 h with overpotential lower than 0.05 V and uniform Na metal anodes with no dendritic growth for GPE (Figure 8A). Na metal anode and TiS_2_ cathodes with LE and GPE were also constructed, showing decaying capacity after 300 cycles for LE and gradually increasing capacity for GPE over 2,560 h (1,000 cycles) up to 321 mAh g^−1^ at 200 mA g^−1^ (Figure 8B). This mixed polymer electrolyte exhibited high ionic conductivity and good electrochemical stability in Na|TiS_2_ cells, proving it to be a viable complex in high energy storage systems.

**FIGURE 9:**
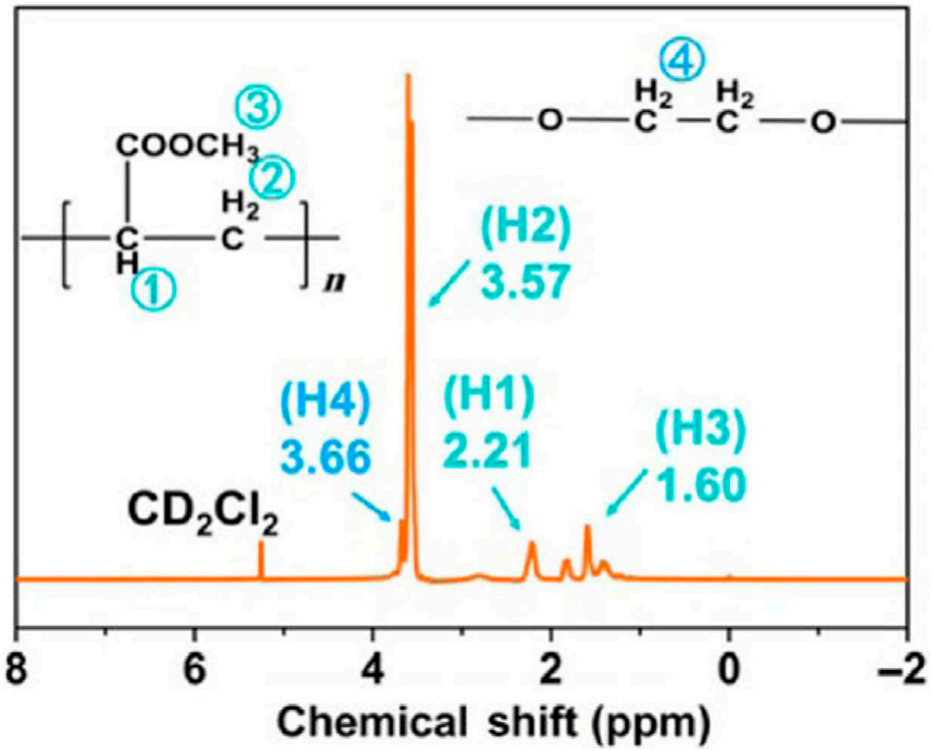
^1^H NMR of CPE. Reproduced with permission from Ref. (Zhang et al., 2018), Nano Research, vol. 11, no. 12, 2018.

Zhang et al., of Nankai University investigated a composite polymer electrolyte (CPE) PEG, poly (methacrylate) (PMA), α-Al_2_O_3_, and NaClO_4_ (Zhang et al., 2018). PEG is mostly responsible for cation transport, while PMA acts as a film-forming agent and promotes salt dissociation, and α-Al_2_O_3_ acts as a filler to reduce crystallinity. Through ^1^H NMR measurements, the composition of CPE was confirmed (Figure 9). EIS measurements showed an ionic conductivity of the CPE ∼10^−4^ S cm^−1^, and Na|Na symmetrical cells exhibited reversible capacity of 85 mAh g^−1^ at 0.5°C and 94.1% capacity retention at 350 cycles.

Poly (vinylidene fluoride) (PVDF) is another popular polymer in battery electrolyte systems. Yang et al., combined a novel sodium-poly (tartaric acid) borate (NaPTAB) salt with PVDF-hexafluoropropylene (PVDF-HFP) to form NaPTAB-SM which is then swelled in a propylene carbonate (PC) to form a GPE (Yang et al., 2020b). Na|Na symmetric cells were constructed using this GPE to test cycling performance. NMR measurements of ^1^H and ^11^B sites were used to characterize structures, and solid state measurements of ^23^Na and ^19^F sites were used to investigate electrolyte chemistry during battery cycling. EIS measurements show an ionic conductivity ∼10^−4^ S cm^−1^ between 30°C and 60°C and a transference number of 0.91. Cycling performance tests of Na|Na symmetric cells shows a capacity of 45 mAh g^−^1 after 500 cycles with high Coulombic efficiency (>98%).

**FIGURE 10:**
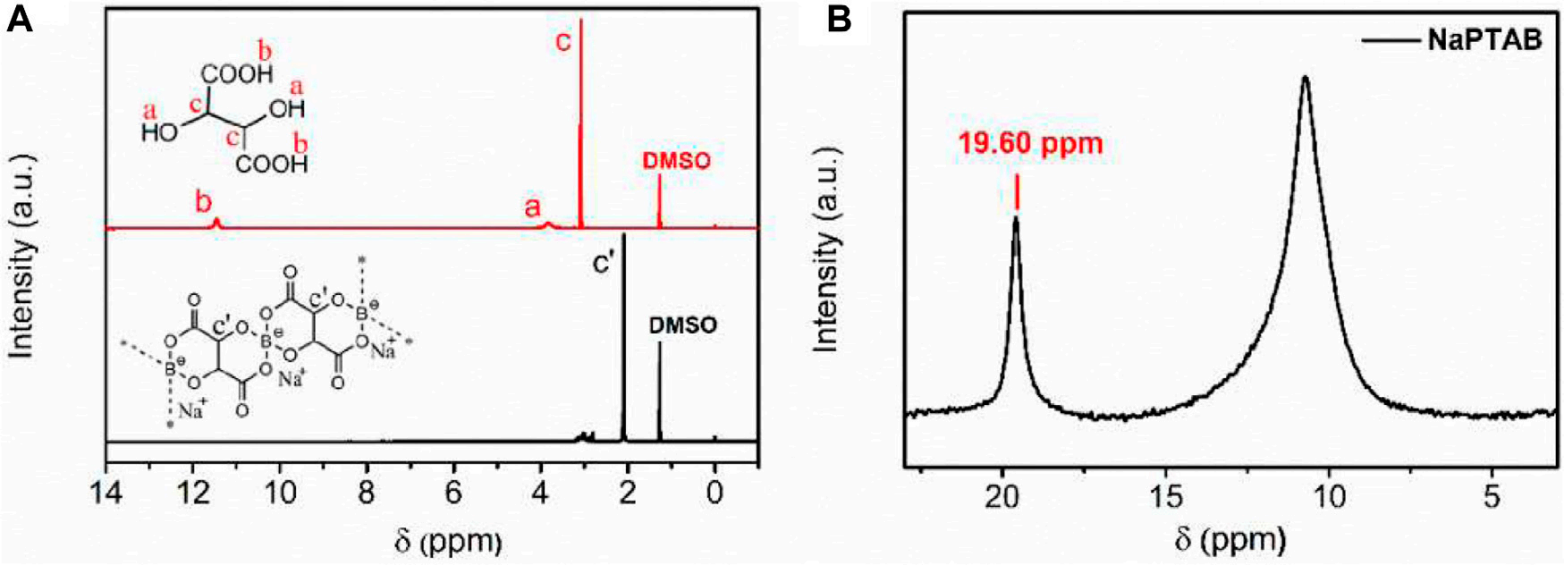
**(A)** ^1^H NMR spectra of NaPTAB and tartaric acid in DMSO-d_6_. **(B)** ^11^B NMR spectra of NaPTAB in DMSO-d_6_ with H_3_BO_3_ signal at 19.60 ppm. Reproduced with permission from Ref. (Yang et al., 2020b), ACS Applied Energy Materials, vol. 3, no. 10, 2020.

**FIGURE 11:**
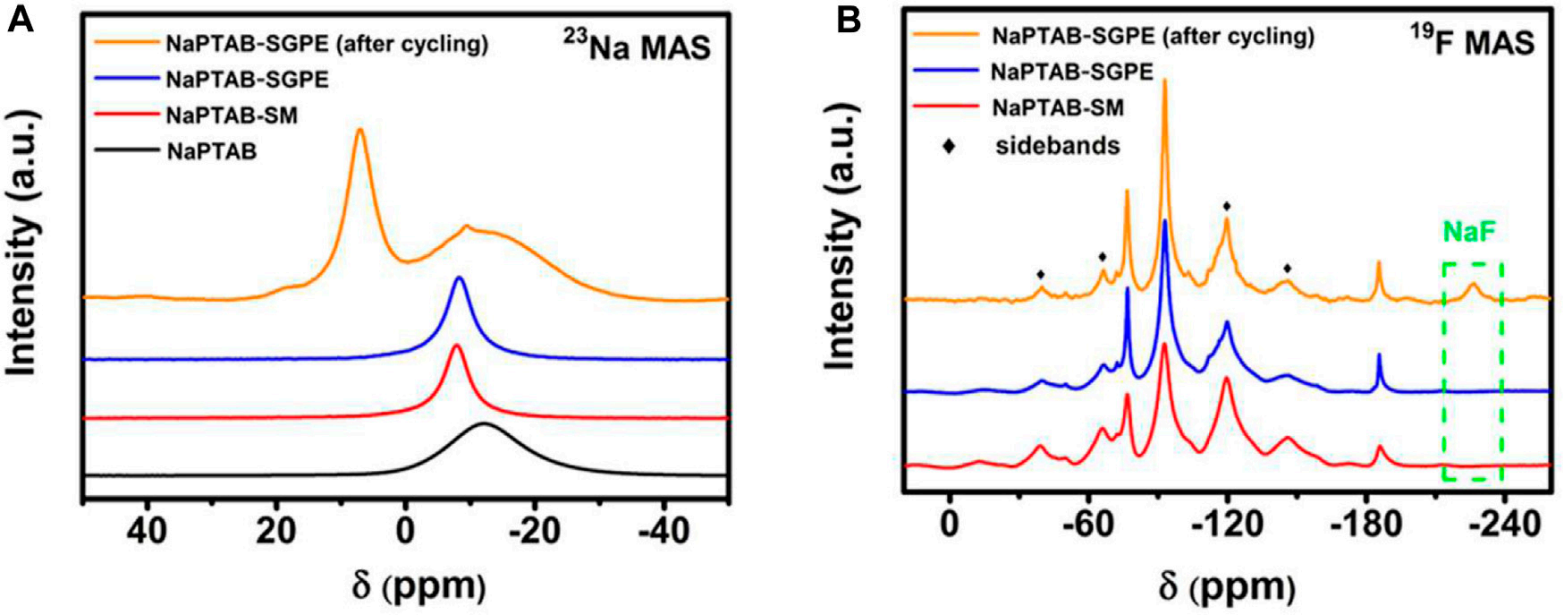
**(A)** ^23^Na MAS NMR and **(B)** ^19^F MAS NMR spectra of NaPTAB, NaPTAB-SM, NaPTAB-SGPE before and after cycling. Reproduced with permission from Ref. (Yanget al., 2020b), ACS Applied Energy Materials, vol. 3, no. 10, 2020.

The ^1^H NMR results show -OH and -COOH groups disappear after reaction and a chemical shift in the -CH group indicating formation of polymeric borate (Figure 10A). The ^11^B NMR results confirm the formation of polymeric borate as a single chemical structure (Figure 10B). The ^23^Na NMR results show a single peak whose linewidth narrows when NaPTAB is mixed with PVDF-HFP, indicating higher mobility of sodium ions (Figure 11A). After 150 cycles, this peak shifts to a high field and broadens, and two new peaks form at 7.10 ppm and 19.7 ppm, indicating new structures generated during cycling. The 7.10 ppm peak indicates NaF in the SEI developed during cycling. The ^19^F NMR results confirm this formation of NaF after cycling with a new peak at −226 ppm after 150 cycles (Figure 11B).

Xie et al., developed a flexible GPE using PVDF-HFP base with water-insoluble N-methyl-N-butyl piperidine difluoromethylimide [(PP_14_)(TFSI)] IL with a polydopamine coating (Xie et al., 2019). PVDF-HFP are well-established as mechanically, chemically, electrochemically, and thermally stable host polymers for electrolyte systems, thought they lack in ionic conductivity. ILs provide excellent ionic conductivity due to favorable ion transport mechanisms yet are highly viscous. The combination of stable, porous GPE, PVDF-HFP, a IL plasticizer, [PP_14_][TFSI], and a thin hydrophilic coating of polydopamine to provide improved wettability and interfacial adhesion was tested in a sodium-ion button battery system using a Prussian blue cathode and metallic sodium anode.

**FIGURE 12:**
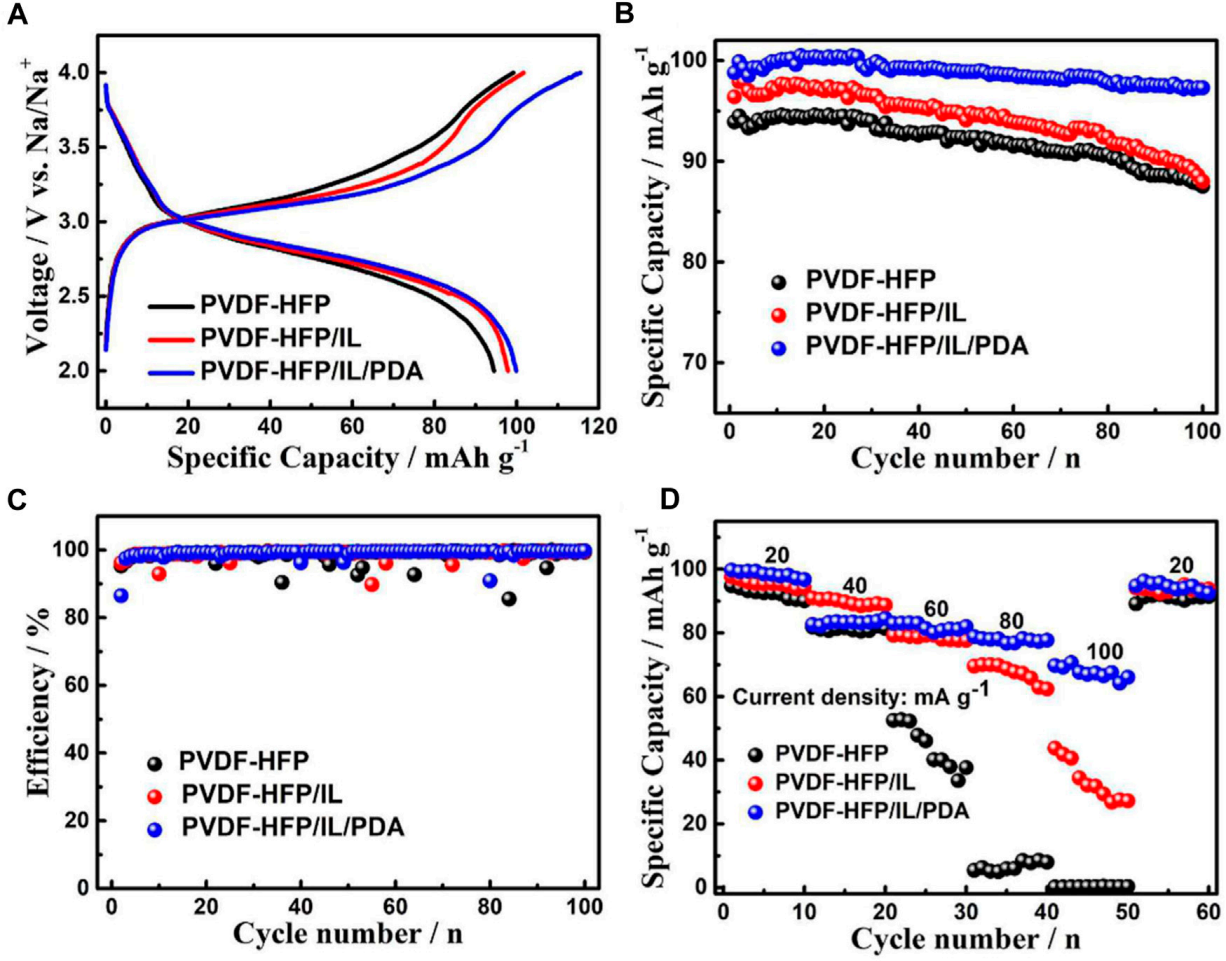
**(A)** Initial charge/discharge curves at current density 20 mA g^−1^. **(B)** Cycle performance at current density 20 mA g^−1^. **(C)** Coulombic efficiencies at current density 20 mA g^−1^. **(D)** Rate capability at current densities 20 mA g^−1^,40 mA g^−1^, 60 mA g^−1^, 80 mA g^−1^, and 100 mA g^−1^. Reproduced with permission from Ref. (Xie et al., 2019), ChemElectroChem, vol. 6, no. 9, 2019.

Electrochemical performance of test cells was tested by measuring cell voltage and specific capacity. Three batteries were constructed with PVDF-HFP, PVDF-HFP/IL, and PVDF-HP/IL/PDA. Batteries were cycled between 2–4 V, with discharge specific capacities of approximately 94.5, 97.9, and 99.9 mAh g^−1^ for the PVDF-HFP, PVDF-HFP/IL, and PVDF-HP/IL/PDA button batteries, respectively, at a current density of 20 mA g^−1^ (Figure 12A). After 100 cycles, the discharge specific capacity for PVDF-HFP/IL/PDA battery was 97.3 mAh g^−1^, exhibiting a capacity retention of 97.4% (Figure 12B). This capacity retention was significantly higher than that of the other two systems. All three systems exhibited high-capacity retention, with PVDF-HFP exhibiting 92.6% and PVDF-HFP/IL exhibiting 89.9% capacity retention (Figure 12C). The rate capability of these battery systems was tested at various discharge current densities, with PVDF-HFP/IL/PDA exhibiting excellent discharge specific capacity stability at high discharge rates (Figure 12D). For all three systems, specific capacity recovered almost completely when the discharge rate was later lowered back to 20 mA g^−1^, indicating no damage to cells. These results indicate the addition of an IL plasticizer and a PDA coating to a GPE matrix greatly improves long term battery performance, likely in part due to the improved interfacial stability attributed to the PDA coating assisting in the formation of a high-quality SEI. Other EIS experiments indicate a thick SEI, confirming the cause of cycle stability, though at the sacrifice of higher charge transfer resistance, reducing specific capacity and polarization.

Genier et al., developed a solid polymer electrolyte based on semi-interpenetrating polymer networks (semi-IPNs) (Genier et al., 2022). Boron-centered polytetrahydrofuran (B-PTHF) and hexamethylene diisocyanate (HMDI) cross-linker were dissolved in N-methyl-2-pyrrolidone (NMP) (solution I). Solutions of sodium perchlorate (NaClO_4_) in NMP were prepared with molar concentrations of B-PTHF chosen so O/Na ratios would be 5, 10, and 30 (solution II). PVDF and B-PTHF solutions mixed with NMP were also formed (solution III). Solutions I-III were then mixed together, and this process was repeated using carbon-centered PTHF (C-PTHF) instead of B-PTHF.

EIS measurements show ionic conductivities of ∼10^−5^ S cm^−1^ for electrolytes with B-PTHF with a ratio of O/Na of 5, while electrolytes with B-PTHF with a ratio of O/Na of 10 and 30 and electrolytes with C-PTHF with a ratio of O/Na of 5, 10, and 30 show ionic conductivities of ∼10^−7^ S cm^−1^. For B-PTHF, higher salt concentration likely leads to looser coordination of Na^+^ ions, while for C-PTHF, lower level of dissociation due to weaker Lewis acidity of C-O_3_ centers and lower amorphousness likely leads to lower conductivities. The transference number for B-PTHF 10 was 0.884 and 0.775 for C-PTHF.

**FIGURE 13:**
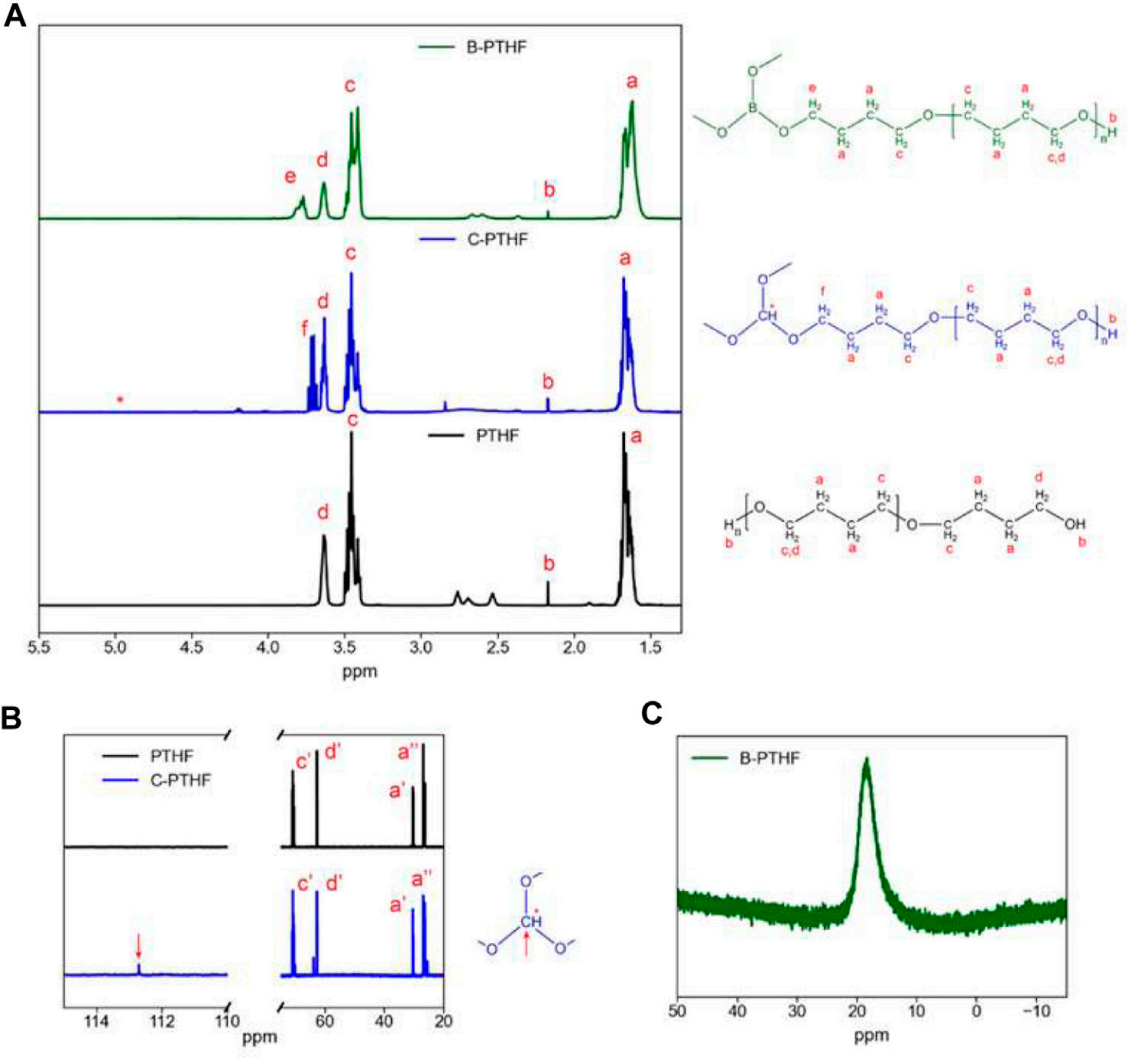
**(A)** ^1^H NMR spectra of PTHF, C-PTHF, and B-PTHF. **(B)** ^13^C NMR spectra of PTHF and C-PTHF. **(C)** ^11^B NMR spectra of B-THF. CDCl_3_ was used as solvent. Reproduced with permission from Ref. (Genier et al., 2022), ACS Applied Polymer Materials, vol. 4, no. 10, 2022.

^1^H, ^11^B, and ^13^C, NMR measurements were used to characterize the structure of B-PTHF and C-PTHF. The ^1^H NMR results show new signals in B-PTHF and C-PTHF not present in PTHF corresponding to protons closer to boron or carbon centers (Figure 13A). A signal associated with the tertiary C-H is not seen in C-PTHF ^1^H NMR spectra due to the proton’s low concentration, so ^13^C NMR comparing PTHF and C-PTHF confirms the reaction (Figure 13B). The ^11^B spectra confirms the single boron center in B-PTHF with a single peak visible, indicating a uniform structure (Figure 13C).

**FIGURE 14:**
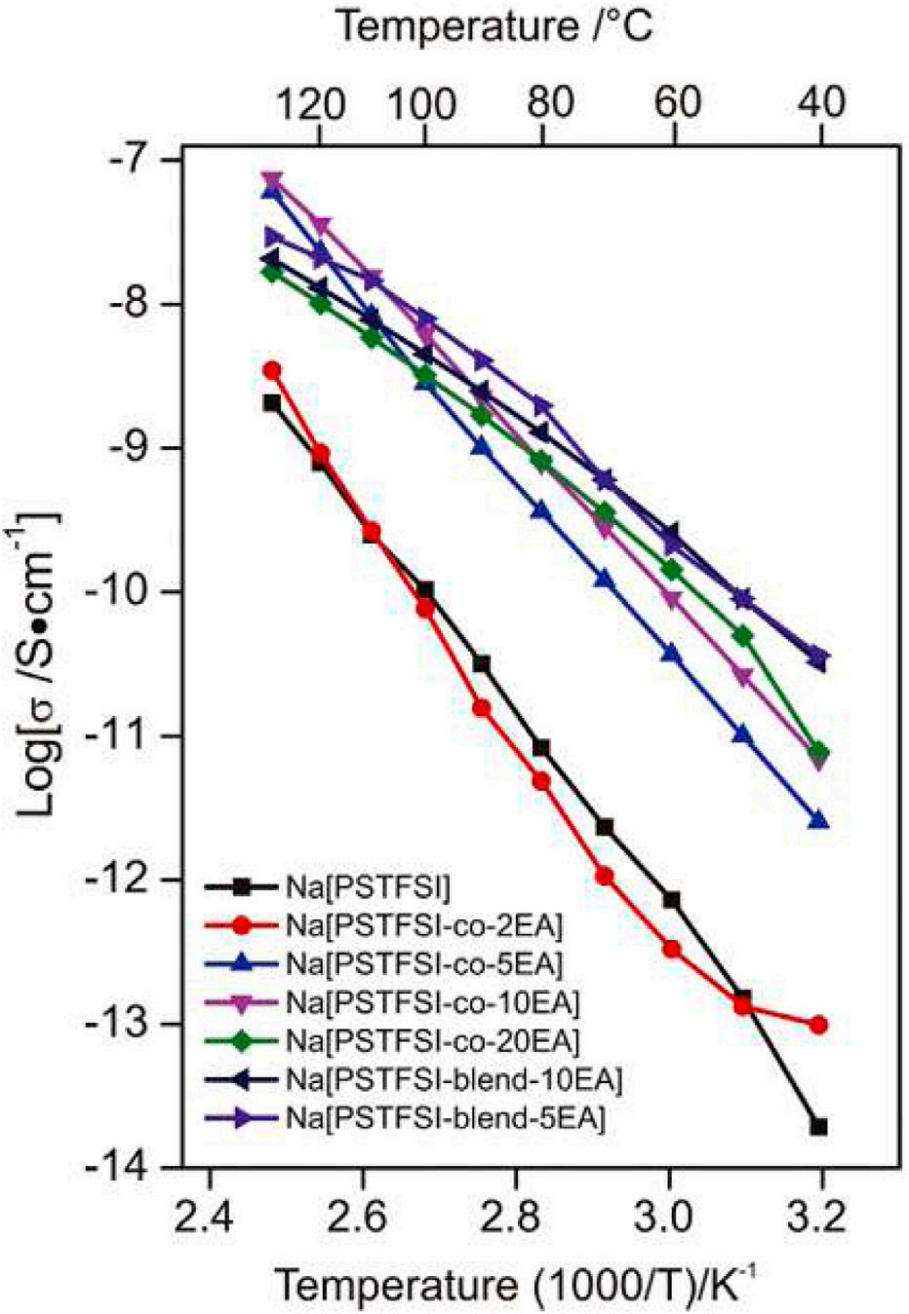
Ionic conductivity of solid polymer electrolytes and blends as a function of temperature. Reproduced with permission from Ref. (Li et al., 2015), Electrochimica Acta, vol. 175, 2015.

Li et al., compared various ratios of sodium 4-styresulfonyl (TFSI) imide [Na (TFSI)] and ethyl acrylate (EA) with the copolymer blend of Na(PSTFSI) and poly (ethylacrylate) (PEA) (Li et al., 2015). AC impedance spectroscopy measurements showed EA polymers exhibited ionic conductivities between ∼10^−12^ to 10^−8^ S cm−1 between 40°C and 120°C (Figure 14). These results indicate an increased ionic conductivity with the incorporation of EA in Na (PSTFSI) and PEA mixtures.

**FIGURE 15:**
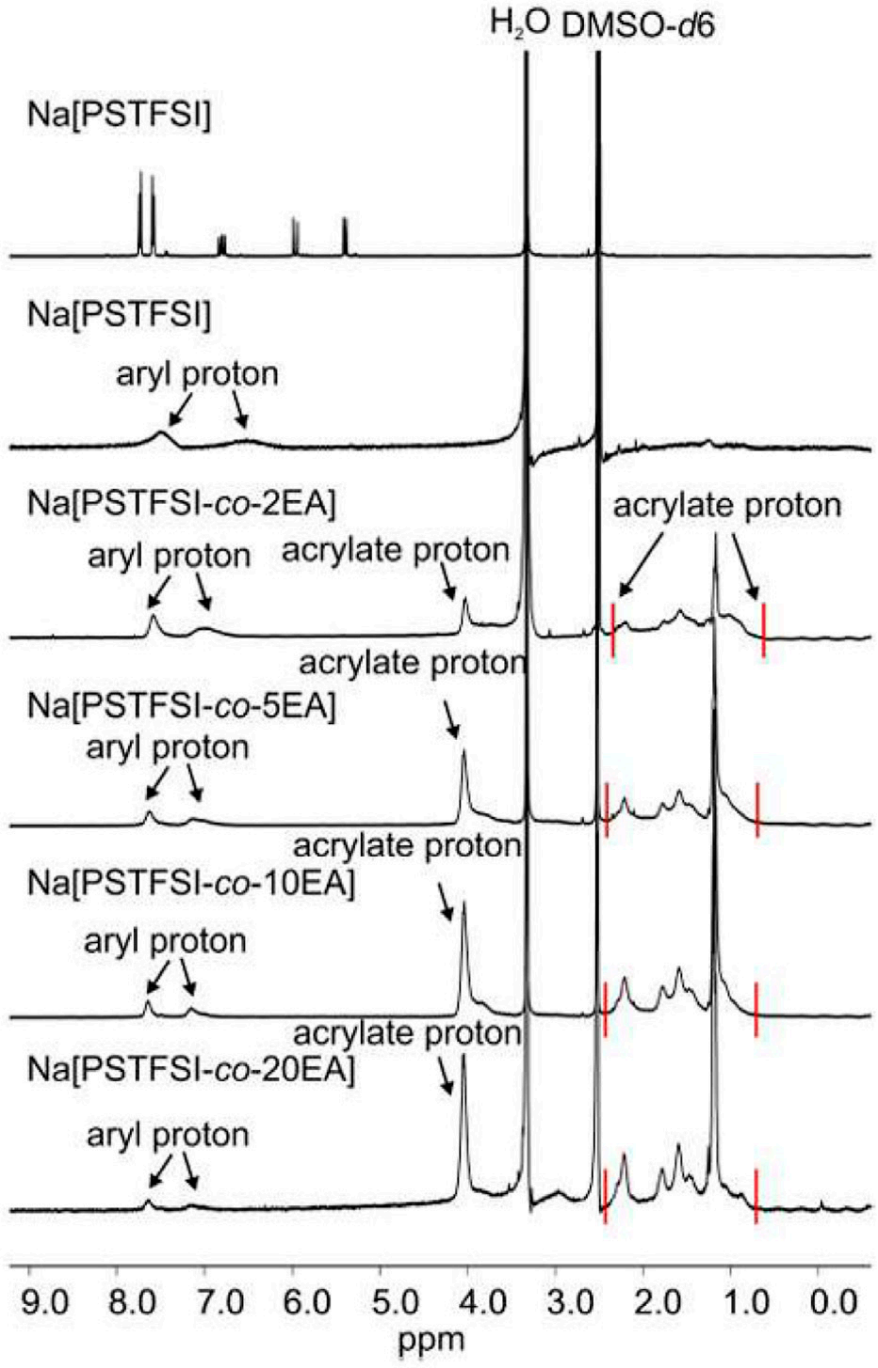
^1^H NMR spectra of Na(PSTFSI) and EA-copolymers. Reproduced with permission from Ref. (Li et al., 2015), Electrochimica Acta, vol. 175, 2015.

^1^H and ^19^F NMR measurements were performed to characterize the structure of polymers. The ^1^H NMR spectra show acrylate proton peaks at 1.0–3.0 ppm and about 4.0 ppm and aryl protons associated with Na^+^ at 7.0–8.0 ppm (Figure 15). By comparing integrals of the aryl proton peak at 7.8 ppm and the acrylate proton at 4 ppm, the ratio of Na^+^ to EA can be estimated. These ratios are ∼1.8, ∼4.1, ∼9.0, ∼20 for Na (PSTFSI-co-2EA), Na (PSTFSI-co-5EA), Na (PSTFSI-co-10 EA), and Na (PSTFSI-co-20EA), respectively, with an error of at least ±10%. This indicates that EA is not fully polymerized, likely due to a difference in reactivity ratios. The ^19^F spectra indicated a single peak at ∼ 78 ppm and is not included in this paper.

### Glyme-based electrolytes

**FIGURE 16:**
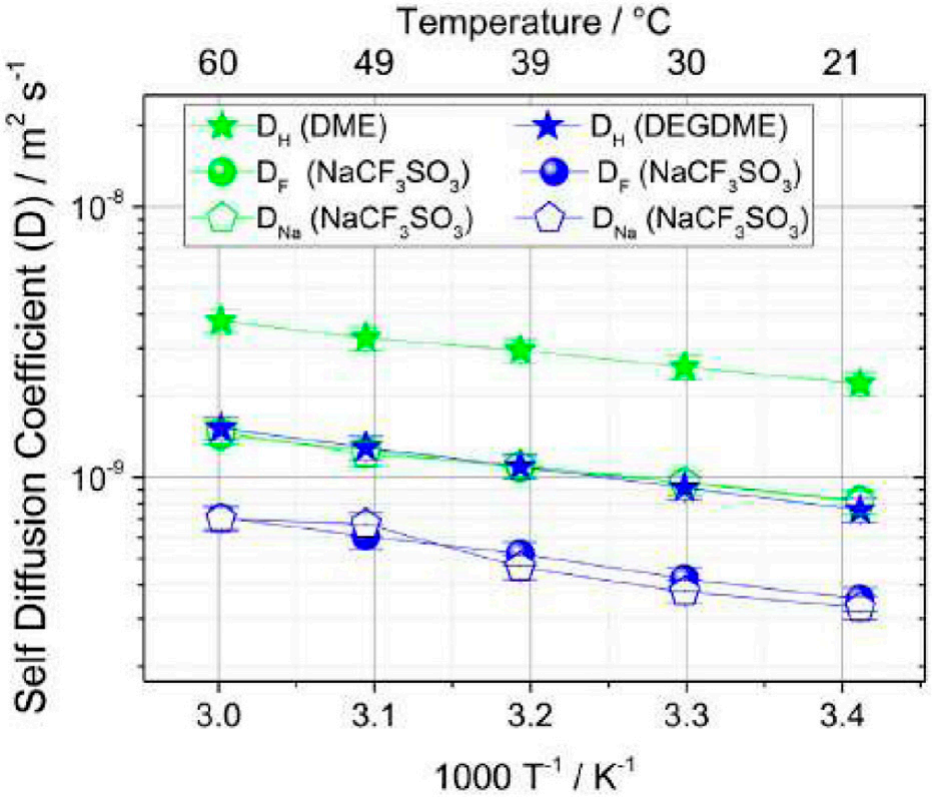
Self-diffusion coefficients of 1H, 19F, and 23Na for DME-NaCF_3_SO_3_ and DEGDME-NaCF_3_SO_3_ from 20°C to 60°C. Reproduced with permission from Ref. (Carbone et al., 2017), Electrochimica Acta, vol. 231, 2017.

Although low molecular mass polyethers such as glymes are liquid at ambient temperature, they have served as a model for PEO-based electrolytes in the context of investigating ion solvation and association. In the case of lithium electrolytes, the glymes have in their own right been extensively studied. The proposed use of glymes as a lithium electrolyte (Carbone et al., 2015) has been adapted for sodium chemistries by Carbone et al., for sodium trifluoromethane sulfonate (NaCF_3_SO_3_) salts dissolved in DME or in diethyleneglycoledimethylether (DEGDME) for a proposed new sodium sulfur cell (Carbone et al., 2017). Transport mechanisms were investigated through self-diffusion measurements from 20°C to 60°C of ^1^H relating to the hydrogen in the glyme chain, ^19^F relating to the fluorine in the CF_3_SO_3_^−^ anion, and ^23^Na relating to the cation (Figure 16).

Both electrolytes show lower ^19^F and ^23^Na self-diffusion coefficients than ^1^H, indicating lower mobility of ions compared to the solvent. DME-NaCF_3_SO_3_ results overall show higher self-diffusion coefficients than DEGDME-NaCF_3_SO_3_, likely due to the lower viscosity of DME with respect to DEGDME. Ionic transference numbers were calculated from the self-diffusion coefficients of ^19^F for the anion and ^23^Na for the cation using the following formula:

$$t^{+}=\frac{D_{Na}}{D_{Na}+D_{F}}$$

Transference numbers for both electrolytes were found to be about 0.5 across the whole temperature range, making the electrolytes viable chemistries for sodium batteries. This value could indicate strong ion pairing, since pair-dominated systems have transference numbers around 0.5. NMR estimate of ion conduction was calculated using NMR-obtained self-diffusion coefficients the Nernst-Einstein equation:

$$\delta_{NMR}=\frac{F^{2}\left[C\right]}{RT}\left(D_{Na}+D_{F}\right)$$

**FIGURE 17:**
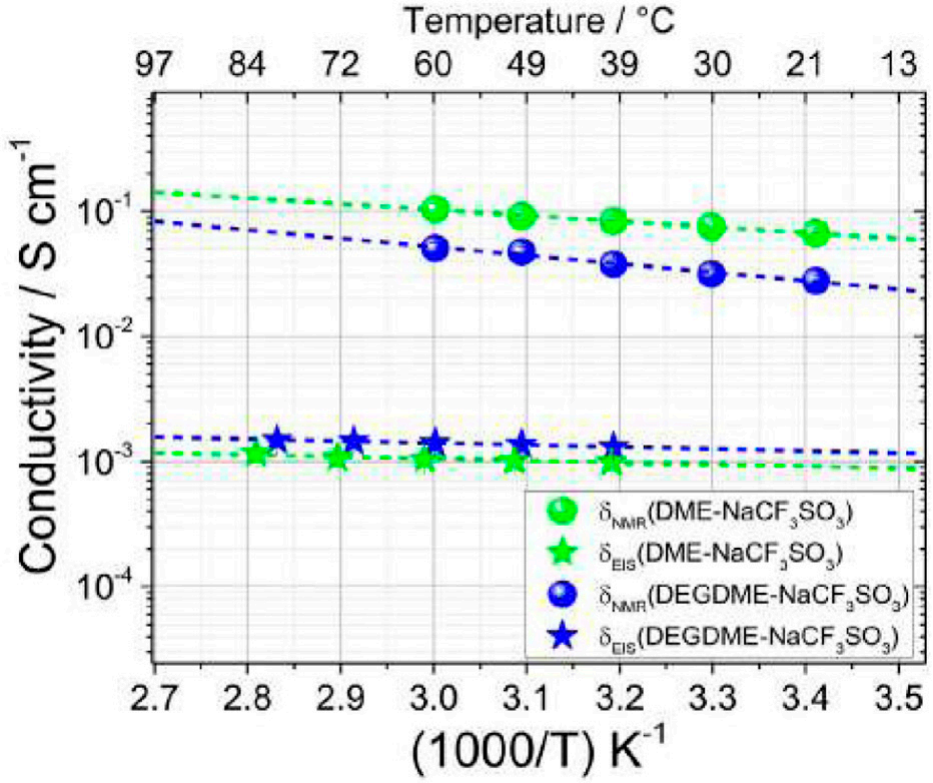
NMR estimate of ion conduction and EIS measured conductivity for DME-NaCF_3_SO_3_ and DEGDME-NaCF_3_SO_3_. Reproduced with permission from Ref. (Carbone et al., 2017), Electrochimica Acta, vol. 231, 2017.

NMR estimates of ion conduction tend to overestimate EIS measured ionic conductivity due to the NMR sensitivity to all ionic species within a solution (including ion pairs or groups), while EIS only considers mobile charge carriers. This is seen when comparing the ion conduction ratios and ionic conductivities for both electrolytes (Figure 17). Overall, both electrolytes demonstrate practical conductivity measured by EIS on the order of 10^−3^ S cm^−1^.

Ionic association was calculated based on the ion conduction ratio values determined by NMR ( $\delta_{NMR}$ ) and the conductivity measured by electrochemical impedance spectroscopy (EIS) ( $\delta_{EIS}$ ) using the following formula:

$$\alpha =\left(1-\frac{\delta_{EIS}}{\delta_{NMR}}\right)$$

Both electrolytes show an increase in ion association with increasing temperature, consistent with other research on polyethers (Carbone et al., 2016). DME-NaCF_3_SO_3_ shows higher overall ion association compared to DEGDME-NaCF_3_SO_3_, indicating a lower ion solvating ability (Kaulgud et al., 2006) of DME.

**FIGURE 18:**
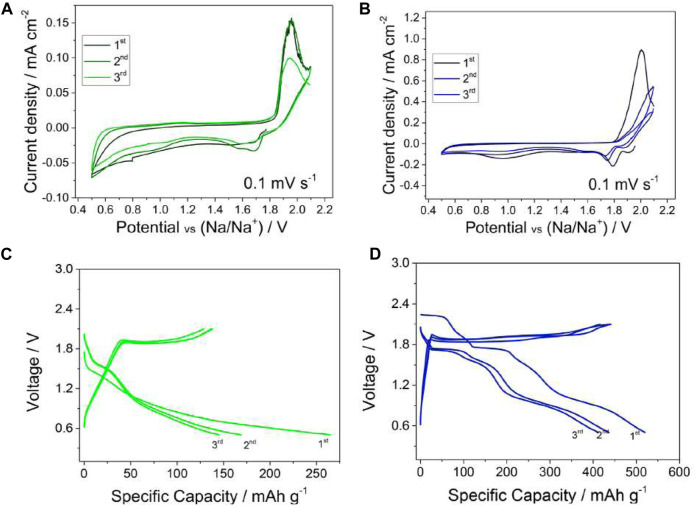
Charge/discharge cycles for sodium-sulfur coin cells for **(A/C)** DME-NaCF_3_SO_3_-NaNO_3_ and **(B/D)** DEGDME-NaCF_3_SO_3_-NaNO_3_. Reproduced with permission from Ref. (Carbone et al., 2017), Electrochimica Acta, vol. 231, 2017.

Sodium-sulfur 2032 coin cells were constructed using the S-MWCNs cathode, a Na-metal anode, and a chosen electrolyte with supplementing NaNO_3_ to form protecting layers at the Na surface. Three charge/discharge cycles were performed on both cells, showing reversible peaks at around 1.8 V, with DEGDME-NaCF_3_SO_3_-NaNO_3_ showing improved intensity and shape, suggesting faster kinetics for the Na/S electrochemical process (Figures 18A, B). For DME-NaCF_3_SO_3_-NaNO_3_, galvanostatic cycles show a specific capacity around 250 mAh g^−1^ during the first cycle with a decrease to around 160 mAh g^−1^ during following cycles. For DEGDME-NaCF_3_SO_3_-NaNO_3_, the specific capacity is around 500 mAh g^−1^ during the first cycle with a decrease to about 450 mAh g^−1^. The DEGDME-NaCF_3_SO_3_-NaNO_3_ electrolyte shows multistep galvanostatic behavior with evolutions at 2.1 V, 1.8 V, and 1.1 V during discharge and a flat 1.9 V during charging (Figures 18C, D). Overall, the characteristics of DME-NaCF_3_SO_3_ and DEGDME-NaCF_3_SO_3_ glyme-based sodium electrolytes show promising results for sodium-sulfur battery applications, with DEGDME chemistry being favored.

**FIGURE 19:**
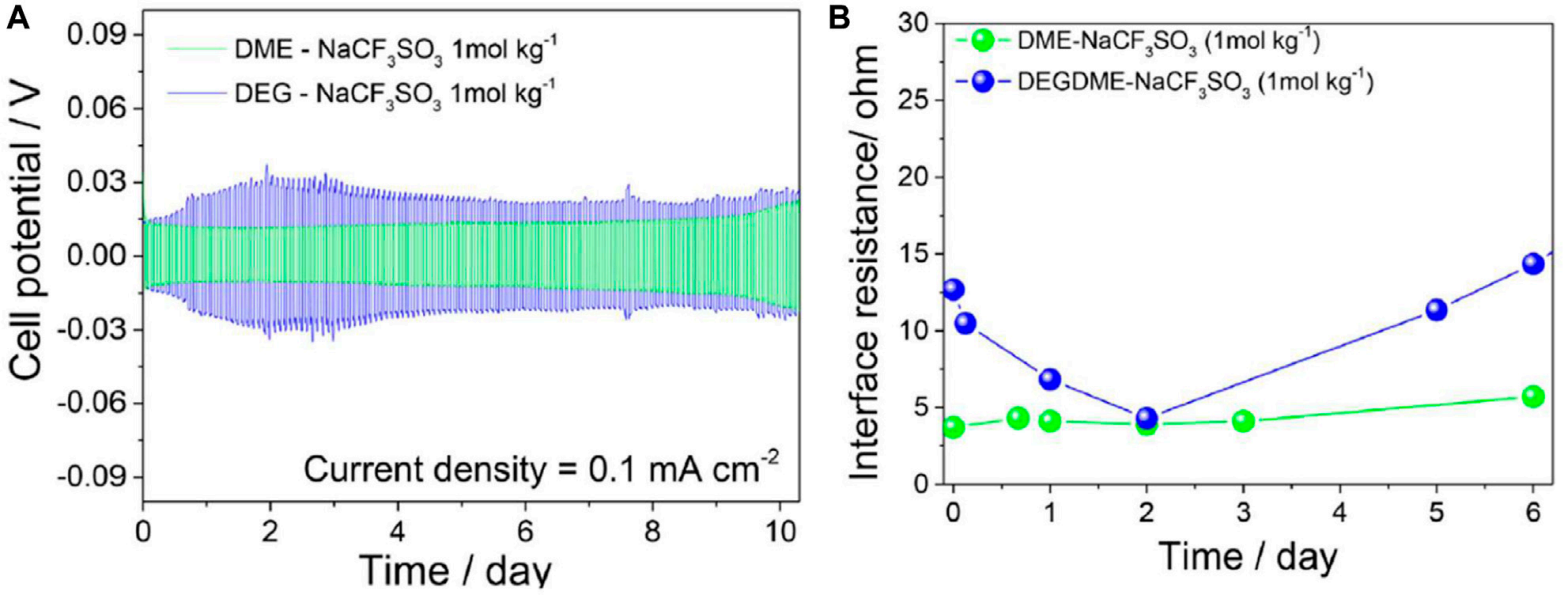
**(A)** Voltage profiles for DME-NaCF_3_SO_3_ and DEGDME-NaCF_3_SO_3_ electrolytes in symmetrical Na/electrolyte/Na cells. Current density 0.1 mA cm^−2^, 1 h of step time limit. **(B)** EIS measurements performed with 10 mV signal amplitude between 0.1 Hz–0.5 MHz frequency range for DME-NaCF_3_SO_3_ and DEGDME-NaCF_3_SO_3_ electrolytes in symmetrical Na/electrolyte/Na cells. Reproduced with permission from Ref. (Carbone et al., 2017), Electrochimica Acta, vol. 231, 2017.

Electrode interfacial chemical stability *versus* over time was studied by EIS in a symmetrical Na/Na 2032 coin-cell at a 10 mV signal amplitude between 0.1 Hz–0.5 MHz frequency range. Battery constructions with DME and DEGDME-based electrolytes were compared. Over the course of 10 days, cell polarization stabilized around 15 mV for DME-based electrolyte and 30 mV for DEGDME-based electrolyte (Figure 19). EIS revealed interfacial resistances lower than 15 Ω for both samples, with lower and more stable resistance trends for DME-based electrolyte. Cell potential polarization and interfacial resistance trends were due to repeated SEI layer dissolution and reformation. These results show the stability and viability of these electrolytes, particularly DME-NaCF_3_SO_3_ electrolytes with sodium metal electrodes.

**FIGURE 20:**
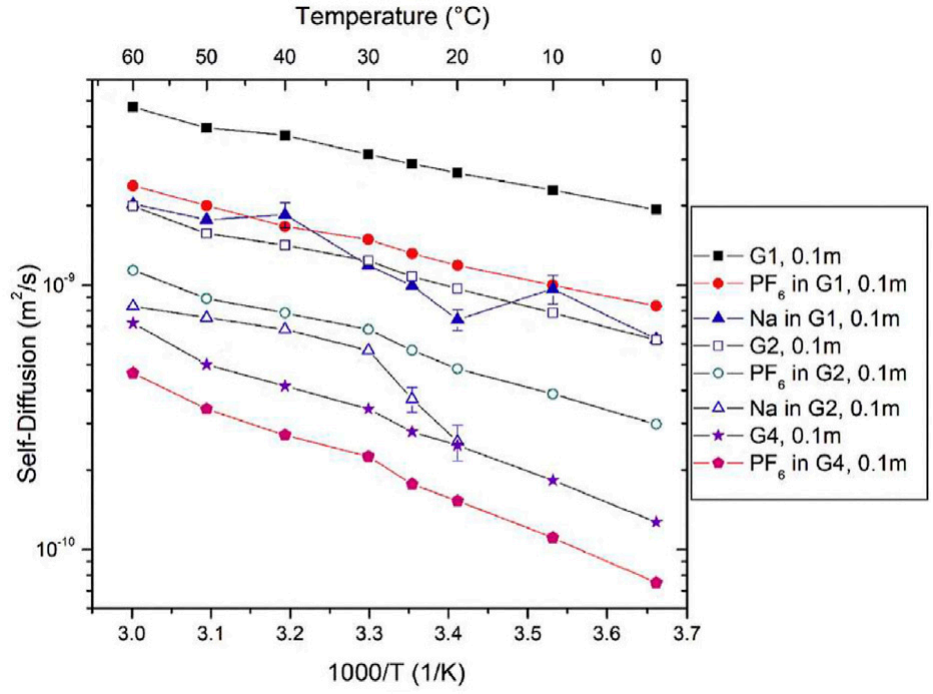
Self-diffusion coefficients of 0.1 m NaPF_6_ as a function of temperature. Reproduced with permission from Ref. (Morales et al., 2019), Electrochimica Acta, vol. 304, 2019, pp. 239–245.

Morales et al., studied NaPF_6_ and LiPF_6_ salts in Monoglyme (G1), Diglyme (G2), and tetraglyme (G4) using similar NMR analysis to the previous work (Morales et al., 2019). Self-diffusion coefficients were measured using PFG NMR for ^1^H corresponding to glymes, ^19^F corresponding to PF_6_ anions, and ^23^Na corresponding to cations between 0°C and 60°C in 0.1 m NaPF_6_ salts in G1, G2, and G4 glymes (Figure 20). Due to the quadrupolar nature of sodium as previously discussed, D_Na_ could not be attained for Na in G2 glymes at low temperature or for Na in G4 glymes across the entire temperature range. Solvent mobility is significantly higher than ion mobility, as seen by the higher ^1^H self-diffusion coefficients compared to those of ^19^F and ^23^Na. Higher self-diffusion coefficients in G1 are likely due to higher mobility in a less viscous solvent.

**FIGURE 21:**
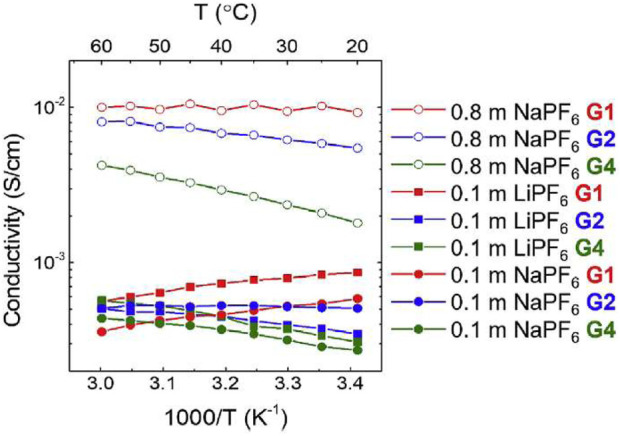
Ionic conductivity of 0.8 m NaPF_6_, 0.1 m NaPF_6_, and 0.1 m LiPF_6_ in G1, G2, and G4 glymes as a function of temperature. Reproduced with permission from Ref. (Morales et al., 2019), Electrochimica Acta, vol. 304, 2019, pp. 239–245.

NMR estimates of ion conductivity were compared to EIS measured ion conductivity (Figure 21). Conductivity was on the order of 10^−2^ S cm^−1^ for NaPF_6_ concentrations of 0.8 m, and on the order of 10^−3^ S cm^−1^ for concentrations of 0.1 m. For 0.8 m NaPF6, strength of temperature dependence of ionic conductivity increases with increased chain length due to strongly temperature-dependent ion association. Ion association is about 0.45, indicating correlated ion motion and increases with temperature due to the decrease of solvent dielectric constant at higher temperature resulting in greater ion attraction. Ion association decreases and ionic conductivity increases with increasing glyme chain length, consistent with other studies.

NaPF_6_ in G1 exhibits decreasing conductivity with temperature and high ionic association, suggesting poor solvation. NaPF_6_ in G4 exhibits better conductivity ionic association properties but could present issues due to high viscosity. NaPF_6_ in G2 has a lower ionic association than G1, and similar and increasing conductivity as temperature increases, indicating G2 could be the most viable glyme of the three for sodium electrolyte systems. Interestingly, the link between the temperature dependence of the host dielectric constant and ion association in high molecular mass polyethers was reported over 30 years ago (Greenbaum et al., 1990).

Ma et al., developed a quasi-solid sodium polymer electrolyte based on NaTFSI and DOL with 20% volume ratio of fluoroethylene carbonate (FEC) with a Al (OTf)_3_ (Ma et al., 2023). These electrolytes were termed “PDFE-5” and “PDFE-20,” representing polymerized 1 M NaTFSI/DOL-FEC (95:5, v:v) and 1 M NaTFSI/DOL-FEC (80:20, v:v), respectively, with the addition of a Al(OTf)_3_ initiator. Liquid DOL electrolyte (LDE) represents liquid 1 M NaTFSI/DOL electrolyte. Liquid FEC electrolyte (LFE) represents polymerized 1 M NaTFSI/FEC electrolyte. Poly (DOL) electrolyte (PDE), represents 1 M NaTFSI/DOL electrolyte prepared by the addition of 0.5 mM Al(OTf)_3_ initiator. PDE alone is crystalline, while PDFE-20 is transparent with limited flow, and PDFE-5 exhibits an intermediate structure with partial crystallinity. FEC acts as a plasticizer, reducing crystallinity of poly (DOL) and contributing to improved ion transport.

**FIGURE 22:**
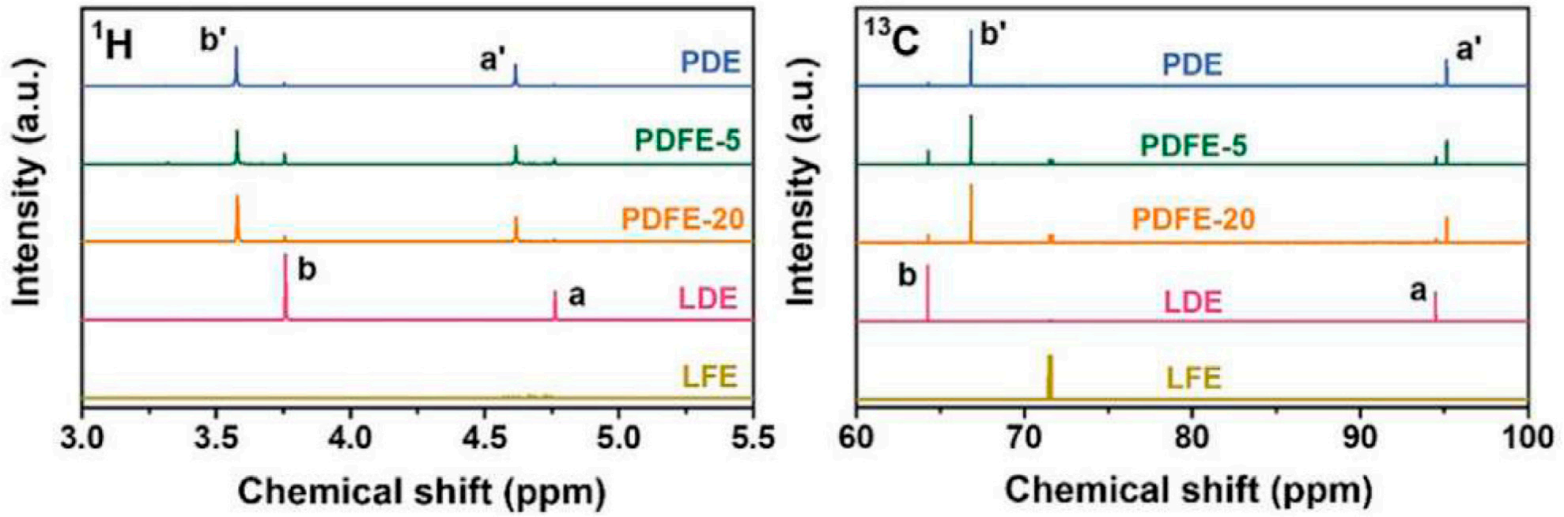
^1^H and ^13^C 1D NMR spectra for PDE, PDFE-5, PDFE-20, and LDE and LFE baseline electrolytes. Labels a and b represent peaks for baseline LDE electrolyte, and labels a’ and b’ represent corresponding peaks for electrolytes containing DOL. The relative chemical shift of these peaks indicate polymerization of DOL, while no chemical shift in the LFE ^13^C peak in samples containing FEC indicates no polymerization of FEC. Reproduced with permission from Ref. (Ma et al., 2023), Journal of Energy Chemistry, vol. 77, 2023.

1D NMR measurements were performed for LDE and LFE baseline electrolytes, PDE, PDFE-5, and PDFE-20. PDE, PDFE-5, and PDFE-20 for ^1^H and ^13^C nuclei to observe DOL and FEC behavior, respectively (Figure 22). Proton peaks exhibit chemical shifts in signals due to polymerization and formation of poly (DOL) structure. Small peaks attributed to liquid DOL are still present, indicating incomplete polymerization of DOL. The ratio of polymerized DOL to total DOL content can be estimated by integration of peak areas for ^1^H NMR spectra. PDE exhibits a ratio of polymerized DOL to total DOL of 95.4%, while PDFE-5 and PDFE-20 exhibit 83.6% and 91.7%, respectively. This is due to the plasticizing effect of FEC, contributing to overall sodium transport. The 72.13 ppm ^13^C NMR peak is present with no chemical shift in all samples containing FEC, indicating that FEC remains in a liquid state across all samples.

**FIGURE 23:**
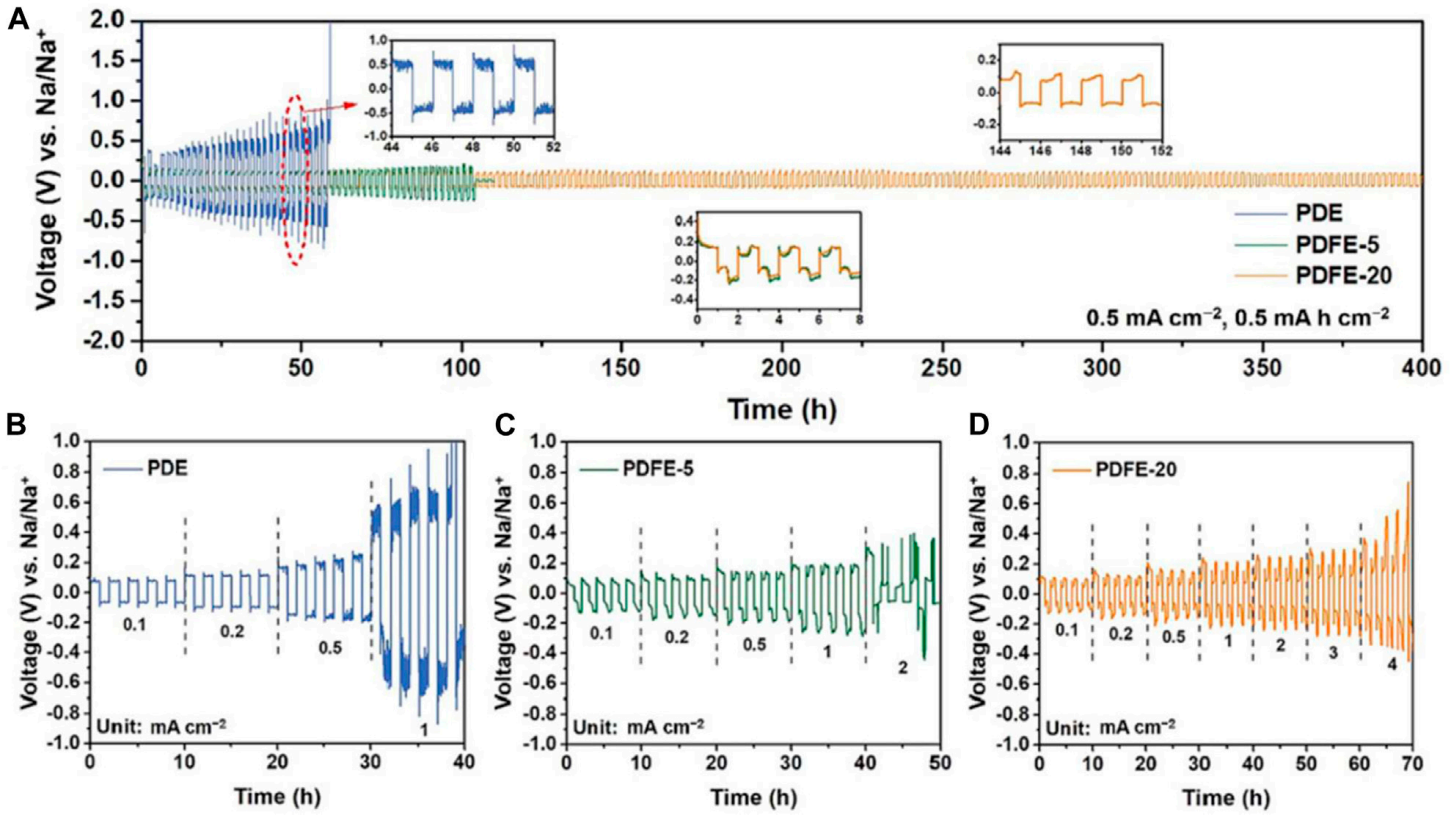
**(A)** Cycling performance for PDE, PDFE-5, and PDFE-20 at current density of 0.5 mA cm^−2^ **(B–D)** Cycling performance at increasing current densities, with five charge/discharge cycles per current. Reproduced with permission from Ref. (Ma et al., 2023), Journal of Energy Chemistry, vol. 77, 2023.

Electrochemical performance was tested in symmetrical Na/Na cells to determine interfacial stability. PDE, PDFE-5, and PDFE-20 were cycled at a current density of 0.5 mA cm^−1^ (Figure 23A). PDE exhibited large overpotential (>0.3 V), eventually leading to a short circuit at 57 h caused by dendritic formation. PDFE-5 experienced a short circuit at 105 h, while PDFE-20 experienced stable performance at lower overpotential (<0.15 V) for over 400 h. PDE, PDFE-5, and PDFE-20 also underwent cycling performance tests at increasing current densities with five charge/discharge cycles per current (Figures 23B–D). PDFE-20 exhibits significant improvement of cycle stability at higher current densities, indicating formation of stable SEI, likely rich in NaF, contributing to desirable interfacial performance.

## Conclusion

There is a growing need to develop alternatives to LIBs in the effort to transition from fossil fuels to renewable energy. Concerns of rising energy consumption demands, environmental and socio-economic malpractice, production and maintenance cost, and battery performance, lifetime, and safety related to lithium use in batteries are being addressed. Our future demands lithium-alternative batteries, and sodium electrolytes are proving to be a strong frontrunner in the race to create a world supported by sustainable batteries. Sodium electrolytes can be formed from a wide range of chemistries with potential practical application. NMR continues to be a useful tool in uncovering sodium polymer electrolytes with properties appropriate for the expansive scale that sustainable battery technology must reach. By incorporating NMR experiments with other common tests, researchers are able to directly determine electrolyte and SEI structure using 1D NMR experiments and investigate ion transport properties through linewidth analysis and PFG diffusion measurements. Due to the quadrupolar nature of ^23^Na, special consideration must sometimes be taken to tailor experiments to the often short T_2_’s while maximizing information obtained from NMR experiments. Nonetheless, a plethora of NMR techniques which can target a wide array of nuclei in liquid and solid systems offer many avenues to obtaining this information. While further research and development is required to discover and create sodium-based systems comparable to current lithium-based battery industry standards, important work is being done to clear the fog around an underutilized cation. With the crucial insight that nuclear magnetic resonance can provide on the transport and structural properties in conjunction with other investigative methods, current and future research on sodium polymer and glyme-based electrolyte systems will continue to show promising results for the future of sustainable battery technology.
